# Disseminated cryptococcosis presenting initially as lower limb cellulitis in a renal transplant recipient – a case report

**DOI:** 10.1186/s12882-018-0815-7

**Published:** 2018-01-27

**Authors:** Katrina Chakradeo, Y. Y. Paul Chia, Cheng Liu, David W. Mudge, Janath De Silva

**Affiliations:** 1 Department of Medicine, Mackay Base Hospital, Bridge Road, Mackay, Australia; 2 Griffith University School of Medicine, Griffith University, Gold Coast, Australia; 3 College of Medicine and Dentistry, James Cook University, Townsville, Australia; 4 Department of Anatomical Pathology, Royal Brisbane and Woman’s Hospital, Brisbane, Australia; 5 Queensland Renal Transplant Service, Princess Alexandra Hospital, Brisbane, Australia

**Keywords:** Case report, Crypotococcosis, *Cryptococcus neoformans*, Cellulitis, Renal transplant

## Abstract

**Background:**

Cellulitis is an unusual presentation of disseminated cryptococcosis, a serious infection seen predominantly in immunocompromised hosts. Disseminated cryptococcosis carries significant morbidity for transplant recipients, especially of the pulmonary and central nervous systems, and carries a high mortality risk.

**Case presentation:**

We report a 59-year-old renal transplant recipient who presented with bilateral lower leg cellulitis without other symptoms or signs. Failure of conventional therapy for cellulitis prompted a skin biopsy confirming cryptococcal cellulitis. Additional evaluation to exclude disseminated disease revealed *Cryptococcus neoformans* in blood cultures and cerebrospinal fluid (CSF). Treatment included reduction in immunosuppression regimen and targeted treatment for cryptococcal disease with liposomal amphotericin B and flucytosine followed by fluconazole consolidation and maintenance therapy. Treatment with liposomal amphotericin B and flucytosine followed by fluconazole consolidation and maintenance therapy achieved a good clinical response. Our patient achieved significant reduction in leg cellulitis and recovered without serious complication.

**Conclusions:**

This case suggests that cutaneous cryptococcosis in immunosuppressed patients warrants a low threshold for investigation for disseminated disease even in the absence of other symptoms or signs.

## Background

*Cryptococcus neoformans* is an encapsulated fungal opportunistic pathogen found in the natural environment and often associated with bird faeces, soil, plants, dust and contaminated food [1, 2]. Cryptococcus can cause serious infections in patients with impaired T cell mediated immunity such as those on immunosuppressant medications [3–5]. *C.neoformans* is usually acquired through inhalation to the lungs and can then disseminate to other sites including the central nervous system (CNS), bone and skin [2, 6, 7]. Disseminated cryptococcosis is an increasingly reported infection in patients with solid organ transplants (SOT) [8] and cryptococcosis accounts for around 8% of invasive fungal infections in SOT recipients following those caused by Aspergillus and Candida [9]. Cryptococcal disease in transplant recipients carries a high mortality risk and this risk is particularly high with disseminated disease, renal failure at baseline and fungaemia [10]. The mortality of cryptococcal CNS disease in transplant recipients is reported as high as 50% [11].

Pulmonary and Central Nervous System (CNS) cryptococcal disease are the most common sites reported for patients with disseminated cryptococcosis [2, 12]. Disseminated cryptococcosis is known rarely to present initially as cellulitis [7, 13–16] and even more unusual for it to present with exclusively lower limb cellulitis without other features to suggest systemic involvement**.**

There is evidence to support that primary cutaneous cryptococcosis (PCC) can occur as a distinct entity [16] and it has been suggested that PCC may serve as a portal of entry for secondary disseminated cryptococcosis [12, 17, 18]. We report a rare case of bilateral lower limb cutaneous cryptococcosis in a patient who lacked evidence of systemic involvement at presentation, with subsequent development and discovery of disseminated cryptococcosis.

## Case presentation

A 59-year-old male retired carpenter received a kidney transplant in August 2014 for end stage renal disease resulting from autosomal dominant polycystic kidney disease and was on maintenance immunosuppression including prednisolone, tacrolimus and mycophenolate. His post-transplant course was complicated by several episodes of urosepsis and graft dysfunction with a baseline serum creatinine of 200 μmol/L and estimated glomerular filtration rate (eGFR) of 29 ml/min. He had recently been treated for CMV disease and *Clostridium difficile* colitis in June 2015. He had no occupational exposures of significance, however kept a pet caged cockatiel for many years which involved regular cleaning of bird droppings from its cage.

He was admitted to hospital firstly on 3rd August 2015. He reported a one-month history of pruritic and painless rash affecting the lower legs bilaterally which had been refractory to self-administered moisturising lotion. On examination he was found to have symmetrical erythematous patches on both shins. He was afebrile throughout the hospital admission and there was no history of fevers or rigors. Nor was there a history of headache, nausea or change in mental state, or any respiratory symptoms. Inflammatory markers (CRP, ESR) were not elevated. During this admission to hospital he received less than 24 h of intravenous (IV) flucloxacillin and was diagnosed with probable lipodermatosclerosis. He was discharged home on 4th August 2015. A superficial skin swab was taken during this admission from a site of mild skin erosion and culture did not isolate a specific micro-organism. He was discharged home on his usual medications including prednisolone, tacrolimus and mycophenolate.

**Fig. 1:**
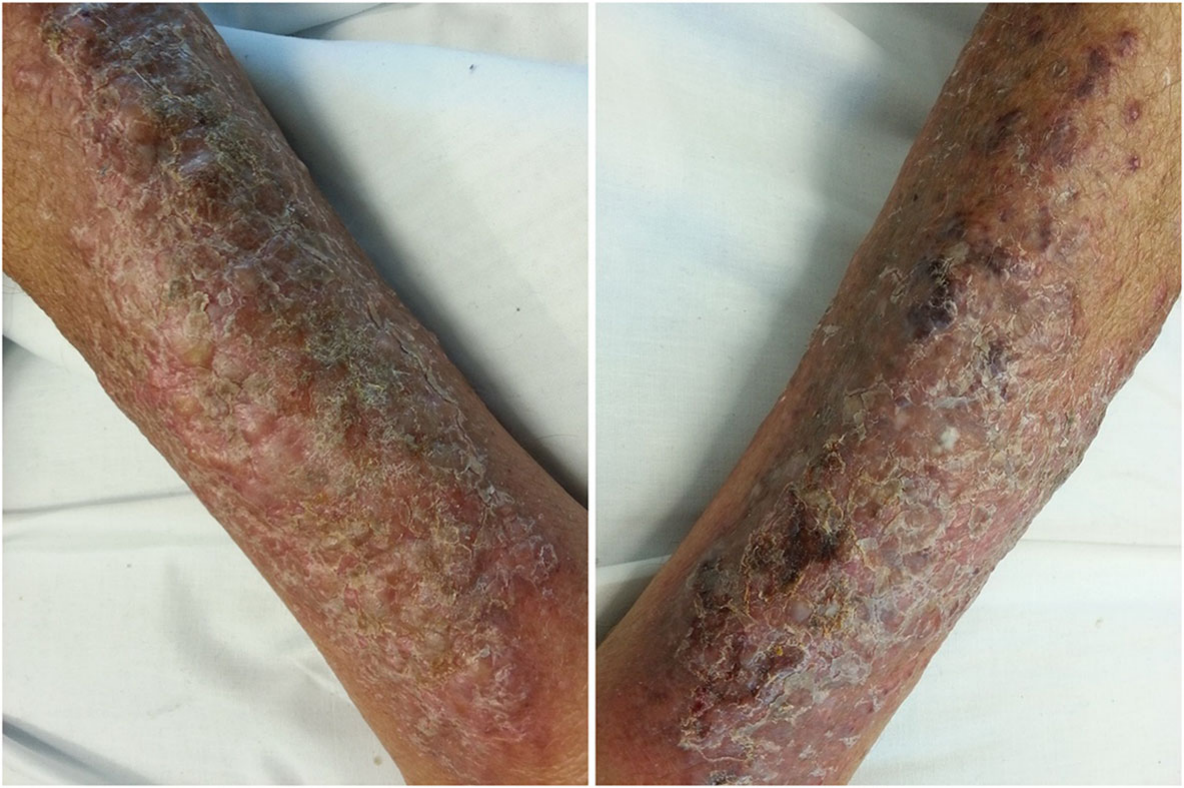
Appearance of lower legs at time of cryptococcal cellulitis diagnosis

**Fig. 2:**
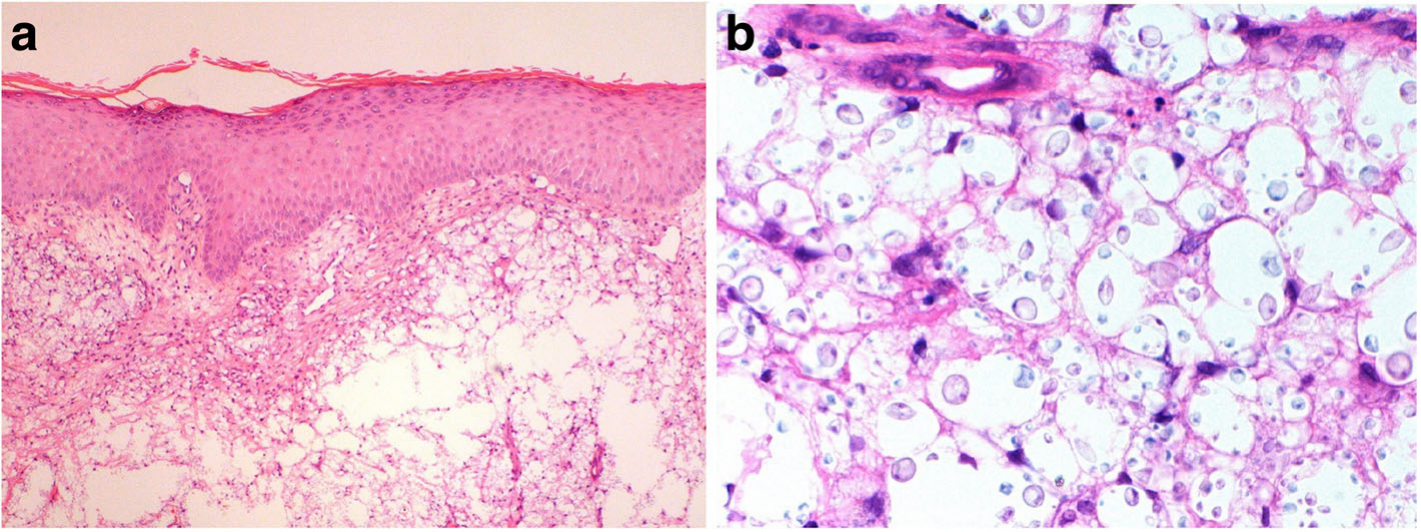
**a** At lower power, the dermis is undermined by fungal organisms while the epidermis is essentially normal (H&E, 40×). **b** Higher power view of the same field demonstrating variably sized ovoid fungi, highly suggestive of *Cryptococcus neoformans* (H&E, 600×)

He represented to hospital on 13th August 2015 with worsening skin rash affecting his lower legs bilaterally (Fig. 1). He had received oral flucloxacillin through his primary care physician for 3 days leading up to this second hospital admission. He was diagnosed initially with venous insufficiency complicated by overlying cellulitis and was initially recommenced on IV flucloxacillin. An infectious diseases physician did not think bacterial cellulitis was likely, given the atypical skin appearance and the presence of bilateral involvement. A dermatology opinion was sought on 14th of August with the preliminary diagnosis of lichen simplex chronicus and suggestion to exclude malignancy and infection. Given the cellulitis was bilateral, persistent, and an atypical presentation, suspicion for an atypical infection and the possibility of systemic aetiology was raised. Skin punch biopsies were performed on the 14th August. Yeast-like organisms highly suggestive of cryptococci were identified on histology (Fig. 2), which on culture were confirmed to be *Cryptococcus neoformans*. Unfortunately sub specification of the organism was not carried out.

Once a diagnosis of cryptococcal cellulitis was made, further investigations were undertaken to determine the extent of cryptococcal disease and determine if systemic infection was present. A computed tomography (CT) scan of the brain was normal. CT of the chest, abdomen and pelvis revealed several non-pathologically enlarged lymph nodes in the mediastinum, retroperitoneum, and bilateral inguinal regions only. Notably there were no discrete pulmonary lesions seen. Lumbar puncture performed on 19th August demonstrated an elevated opening pressure of 28.5 cm of water (reference range up to 20) with white cell count (WCC) 30, Red cell count 1, protein 370 mg/L and glucose 1.5 mmol/L. Unfortunately a serum glucose was not obtained on the same day. Gram staining showed yeast-like organisms and India ink staining of CSF showed organisms resembling Cryptococci. As soon as disseminated cryptococcosis was suspected following initial lumbar puncture, treatment was commenced on the 19th August. The patient was started on renally dose-adjusted IV flucytosine 1500 mg twice daily and IV liposomal amphotericin 200 mg (3 mg/kg) daily after liaison with the nephrology team. His immunosuppression was reduced by withholding mycophenolate on the advice of the transplant physician. Blood and CSF cultures were both found to grow *C.neoformans*. Both CSF and serum cryptococcal antigen titre was > 1:1024. These findings confirmed the diagnosis of disseminated cryptococcal infection with high burden of disease.

Twenty-four hours following commencement on antifungal therapy the patient developed a mild headache without any focal neurological signs. He was transferred to the tertiary transplant centre in Queensland for ongoing management by renal transplant and infectious diseases teams.

A repeat lumbar puncture was performed revealing an opening pressure of 37 cm of water. Ophthalmology review at this time revealed no papilloedema. Further lumbar punctures were continued with repeat lumbar punctures performed on the 24th August, 25th August, 26th August and 2nd September which continued to culture positive for Cryptococci. A final lumbar puncture performed on 29th September showed 8 white cells (100% mononuclear), protein 600 mg, glucose 3.5 mmol/L with a india ink that was positive for Cryptococci however culture was negative.

During hospital treatment for disseminated cryptococcosis our patient developed a concomitant episode of *C.difficile* colitis prompting treatment with oral metronidazole. As a result his kidney function temporarily reduced from a baseline of 27 ml/min to 15 ml/min, which was thought to be related to reduced hydration. This subsequently resolved with rehydration and resolution of *C.difficile* infection. His kidney function remained stable thereafter (baseline eGFR 27 ml/min) on the same regimen of amphotericin and flucytosine. His immunosuppression regimen at the time of *C.difficile* infection included prednisolone and tacrolimus (with mycophenolate being ceased).

He remained in hospital, receiving a total of 6 weeks IV liposomal amphotericin and flucytosine. This induction phase with amphotericin and flucytosine was prolonged (longer than the usual 2 weeks) due to persistence of positive CSF cultures and clinician precaution. He was then changed to oral fluconazole as consolidation and maintenance therapy.

**Fig. 3:**
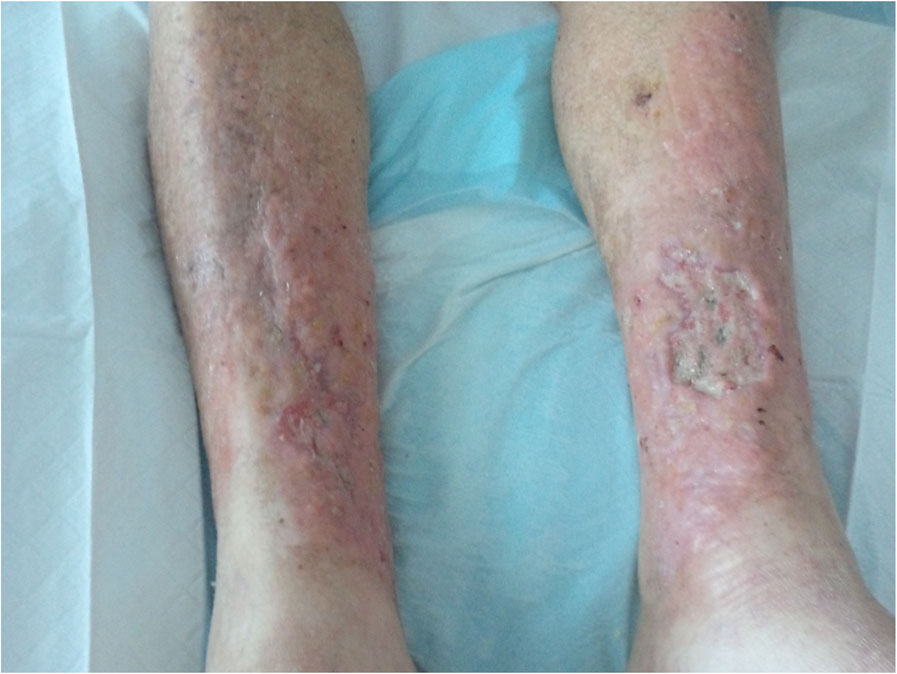
Appearance of legs following treatment

Since this time our patient has seen significant improvement in his lower leg cellulitis (Fig. 3). He no longer has the pet cockatiel bird as this was thought to be a potential source of the initial cryptococcal skin infection. He has not had another hospital admission in relation to his skin disease. His current immunosuppression regimen includes prednisolone and tacrolimus and renal allograft function is stable. He remains on oral fluconazole, and is expected to remain on fluconazole lifelong due to an ongoing need for substantial immunosuppression in the setting of his renal transplant.

## Discussion

*Cryptococcus neoformans* infections predominantly affect immunosuppressed individuals including those with solid organ transplants. In the reported case our patient had received 12 months’ immunosuppression for his renal transplant. Cryptococcosis patients with higher mortality risk are those with baseline renal impairment and disseminated disease such as our case patient [10].

Opportunistic infections such as *Crypotococcus neoformans* are common in renal transplant recipients, especially in the first 6 months following transplantation. Following renal transplants, early infections (less than 1 month) are often associated with the transplant surgical procedure itself or donor derived infections. These include bacterial or candidal wound infections, urinary tract infections, pneumonia, line sepsis, and surgical site infections. Reactivation of HSV can also occur early. In the second month to 6 months post transplant, opportunistic infections such as *Cryptococcus neoformans* are more likely [19, 20]. These can be newly acquired or a reactivation of latent infection. Infections such as CMV, *pneumocystis carinii*, *Aspergillus spp.*, *Nocardia spp.*, *Listeria*, and *Toxoplasmosis gondii* can also be seen. Reactivation of latent infections in transplant recipients can include viral hepatitis, atypicial fungal infections or mycobacterium tuberculosis. From 6 months onwards infections in renal transplant recipients tend to reflect those of the general population and include infections such as influenza, urinary tract infection, and pneumococcal pneumonia. The only common opportunistic pathogen occurring at this time is varicella zoster virus reactivation (herpes zoster) [19]. In our case Cryptococcal infection was diagnosed approximately 1 year after initial transplantation.

Cryptococcal infection in transplant patients can be due to new acquisition or reactivation of latent or dormant infection. For some time cryptococcal infections has been thought to behave with dormancy and reactivation via epidemiological research [21]. Additional research has supported this, showing that *Cryptococcus neoformans* has the characteristics necessary for dormant infection in humans and dormant yeast cells can survive both in vitro and in vivo [22]. In one study of transplant recipients who developed cryptococcosis, 52% of cases showed serological evidence of latent infection prior to transplantation. This study also concluded that these patients developed cryptococcosis earlier than those with new acquisition, at 5.6 +/− 3.4 months. Patients with newly acquired cryptococcosis developed it at a later time, 40.6 +/− 63.8 months post transplantation [23]. This literature suggests it is plausible that our patient may have had either a new infection or latent reactivation of *Cryptococcus neoformans*. However, in a case of reactivation it would be expected that our patient would have had more pulmonary or CNS symptoms at the onset rather than his initial skin manifestation.

Severe cryptococcal infections in immunosuppressed patients most frequently manifest at initial presentation with pulmonary or CNS symptoms [2, 12]. Cryptococcal skin involvement can occur in disseminated disease and is known to be polymorphous in appearance. It can manifest as acneiform papules, ulcers, subcutaneous nodules, and rarely as cellulitis [2, 17]. Classically, haematogenous cryptococcal skin changes are described as umbilicated papules resembling molluscum contagiosum, although any skin lesion is possible [17]. In our case, the skin changes were confined to the lower legs where exposure to cryptococcus from the environment, and in particular his pet cockatiel is plausible. It has been reported before that pet birds are a probable source for disseminated cryptococcal infections from inhaled excreta [18].

Additionally, cellulitis is seldom the initial presenting symptom in disseminated cryptococcal infection as usually disease at this stage involves either the pulmonary or central nervous system. Notably on presentation our patient had no clinical symptoms or signs to suggest either pulmonary or CNS involvement and imaging of the head and chest did not reveal any CNS or pulmonary lesions. CNS involvement in our case was only detected as a result of CSF sampling to screen for disseminated cryptococcosis, upon finding cutaneous cryptococcosis in an immunosuppressed patient. Our patient later developed symptoms associated with CNS disease during systemic anti-fungal treatment that manifested as headache, nausea and mild confusion, and this was short-lived. This acute deterioration in mentation was in association with high CSF opening pressures requiring daily to second daily lumbar punctures initially however an immune reconstitution inflammatory response is also an important consideration in the appropriate clinical setting [12].

There is some evidence within the literature suggesting cutaneous cryptococcal infection may serve as a portal of entry for disseminated disease and this appears to be of particular concern for those who are immunocompromised [7, 13–16]. However, it has been questioned about whether primary cutaneous cryptococcosis (PCC) exists rather than cutaneous cryptococcosis being only secondary to haematogenous dissemination [17]. Whether a potential portal of entry, or marker of disseminated cryptococcosis, the discovery of cutaneous cryptococcus in immunocompromised patients is crucial to enable further investigations and effective management.

This case illustrates the need to investigate inflammatory skin changes promptly in patients who are significantly immunosuppressed such as renal transplant recipients, especially if not responding to conventional treatment. For skin lesions in immunocompromised patients, or cellulitis that does not respond to conventional antimicrobial treatment, a differential diagnosis including atypical opportunistic infections should be considered [24]. This case also demonstrates that the skin may serve as a portal of entry for disseminated disease, and thereby highlights the importance of investigating extensively for disseminated disease in immunosuppressed patients with a diagnosed cryptococcal skin infection even in the absence of systemic symptoms. Delayed diagnosis could be devastating.

Guidelines developed by the Infectious Diseases Society of America (IDSA) recommend amphotericin B used in conjunction with flucytosine as primary induction therapy for disseminated cryptococcosis followed by fluconazole for consolidation and maintenance therapy [25]. It is also recommended that overall immunosuppression is reduced gradually as it is proposed that abrupt withdrawal of immunosuppression could lead to a Th1 pro-inflammatory state thereby increasing risk of immune reconstitution inflammatory syndrome (IRIS) or organ rejection [25, 26]. The balance of reducing glucocorticoids and overall immunosuppression whilst avoiding immune reconstitution or transplant rejection is key. Furthermore, calcineurin inhibitors have direct anti-cryptococcal activity and therefore may be the preferred immunosuppressive agents in those with an ongoing need of immunosuppression such as SOT recipients [25–27]. It has also been shown in vitro that tacrolimus suppresses growth of *C.neoformans* at 37 °C but not at 24 °C suggesting that temperature dependent inhibition of cryptococci may help reduce CNS infection whilst allowing growth of the fungus in more peripheral, cooler sites such as the skin [27, 28]. This exemplifies the importance of suspecting peripheral sites for cryptococcal infections in immunosuppressed patients and supports using calcineurin inhibitors as the preferred choice of immunosuppression in circumstances of cryptococcal infection whereby ongoing immunosuppression is required, such as SOT recipients. Our case received a treatment regimen in accordance with IDSA guidelines achieving a good patient outcome.

## Conclusion

In conclusion, this case demonstrates the importance of timely and accurate diagnosis of cryptococcal disease in immunosuppressed patients in order to facilitate prompt and targeted anti-fungal treatment. It supports the notion that cryptococcal cellulitis in immunocompromised patients may be a portal of entry for disseminated cryptococcal disease and therefore must always be extensively investigated.

## Abbreviations

CNS: Central nervous system
CRP: C-reactive protein
CSF: Cerebrospinal fluid
CT: Computed tomography
ESR: Eosinophil sedimentation rate
IRIS: Immune reconstitution inflammatory syndrome
IV: Intravenous
PCC: Primary cutaneous cryptococcosis
SOT: Solid organ transplant

## References

[1] Srivastava G, Tilak R, Yadav J, Bansal M. Cutaneous cryptococcus: marker for disseminated infection. BMJ Case Rep. 2015; 10.1136/bcr-2015-210898. 10.1136/bcr-2015-210898 PMC4513468 26199299

[2] Valente E, Lazzarin M, Koech B (2015). Disseminated cryptococcosis presenting as cutaneous cellulitis in an adolescent with systemic lupus erythematosus. Infect Dis Rep.

[3] Desnos-Ollivier M, Patel S, Raoux-Barbot D, Heitman J, Dromer F (2015). Cryptococcosis serotypes impact outcome and provide evidence of cryptococcus neoformans speciation. MBio.

[4] Leopold Wager C, Wormley F (2015). Is development of a vaccine against cryptococcus neoformans feasible?. PLoS Pathog.

[5] Sun H, Alexander B, Huprikar S (2014). Predictors of immune reconstitution syndrome in organ transplant recipients with cryptococcosis: implications for the management of immunosuppression. Clin Infect Dis.

[6] Ni W, Huang Q, Cui J (2013). Disseminated cryptococcosis initially presenting as cellulitis in a patient suffering from nephrotic syndrome. BMC Nephrol.

[7] Lu H, Yang Y, Huang Y (2007). Disseminated cryptococcosis initially presenting as cellulitis in a rheumatoid arthritis patient. J Chin Med Assoc.

[8] Neofytos D, Fishman J, Horn D (2010). Epidemiology and outcome of invasive fungal infections in solid organ transplant recipients. Transpl Infect Dis.

[9] Pappas P, Alexander B, Andes D (2010). Invasive fungal infections among organ transplant recipients: results of the Transplant-Associated Infection Surveillance Network (TRANSNET). Clin Infect Dis.

[10] Singh N, Alexander B, Lortholary O (2007). Cryptococcus neoformans in organ transplant recipients: impact of calcineurin-inhibitor agents on mortality. J Infect Dis.

[11] Wu G, Vilchez RA, Eidelman B, Fung J, Kormos R, Kusne S (2002). Cryptococcal meningitis: an analysis among 5,521 consecutive organ transplant recipients. Transpl Infect Dis.

[12] Singh N, Dromer F, Perfect J, Lortholary O (2008). Immunocompromised hosts: cryptococcosis in solid organ transplant recipients: current state of the science. Clin Infect Dis.

[13] Singh N, Rihs J, Gayowski T, Yu V (1994). Cutaneous cryptococcosis mimicking bacterial cellulitis in a liver transplant recipient: case report and review in solid organ transplant recipients. Clin Transpl.

[14] Chaya R, Padmanabhan S, Anandaswamy V, Moin A (2013). Disseminated cryptococcosis presenting as cellulitis in a renal transplant recipient. J Infect Dev Ctries.

[15] Anderson D, Schmidt C, Goodman J, Pomeroy C (1992). Cryptococcal disease presenting as cellulitis. Clin Infect Dis.

[16] Horrevorts A, Huysmans F, Koopman R, Meis J (1994). Cellulitis as first clinical presentation of disseminated cryptococcosis in renal transplant recipients. Scand J Infect Dis.

[17] Neuville S, Dromer F, Morin O, Dupont B, Ronin O, Lortholary O (2003). Primary cutaneous cryptococcosis: a distinct clinical entity. Clin Infect Dis.

[18] Nosanchuk J (2000). Evidence of zoonotic transmission of cryptococcus neoformans from a pet cockatoo to an immunocompromised patient. Ann Intern Med.

[19] Patel R, Paya C (1997). Infections in solid-organ transplant recipients. Clin Microbiol Rev.

[20] Fishman J (2013). Opportunistic infections-coming to the limits of Immunosuppression?. Cold Spring Harb Perspect Med.

[21] Garcia-Hermoso D, Janbon G, Dromer F (1999). Epidemiological evidence for dormant cryptococcus neoformans infection. J Clin Microbiol.

[22] Alaino A, Vernel-Pauilac F, Stury-Leclère (2015). Cryptococcus neoformans host adaptation: toward biological evidence of dormancy. MBio.

[23] Saha D, Goldman D, Shao X (2007). Serologic evidence for reactivation of cryptococcosis in solid-organ transplant recipients. Clin Vaccine Immunol.

[24] Falagas ME, Vergidis PI (2005). Narative review: diseases that masquerade as infectious cellulitis. Ann Intern Med.

[25] Perfect J, Dismukes W, Dromer F (2010). Clinical practice guidelines for the management of cryptococcal disease: 2010 update by the Infectious Diseases Society of America. Clin Infect Dis.

[26] Singh N, Lortholary O, Alexander B (2005). An immune reconstitution syndrome-like illness associated with cryptococcus neoformans infection in organ transplant recipients. Clin Infect Dis.

[27] Husain S, Wagener M, Singh N (2001). Cryptococcus neoformans infection in organ transplant recipients: variables influencing clinical characteristics and outcome. Emerg Infect Dis.

[28] Fox D, Cruz M, Sia R (2001). Calcineurin regulatory subunit is essential for virulence and mediates interactions with FKBP12-FK506 in cryptococcus neoformans. Mol Microbiol.

